# Novel Treatments for PXE: Targeting the Systemic and Local Drivers of Ectopic Calcification

**DOI:** 10.3390/ijms242015041

**Published:** 2023-10-10

**Authors:** Ida Joely Jacobs, Qiaoli Li

**Affiliations:** 1 Biomedical Sciences MS Program, Jefferson College of Life Sciences, Thomas Jefferson University, Philadelphia, PA 19107, USA; ida.joely.jacobs@jefferson.edu; 2 Department of Biochemistry and Molecular Biology, Thomas Jefferson University, Philadelphia, PA 19107, USA; 3 PXE International Center of Excellence in Research and Clinical Care, Thomas Jefferson University, Philadelphia, PA 19107, USA

**Keywords:** ectopic calcification, etidronate, inorganic pyrophosphate, minocycline, pseudoxanthoma elasticum

## Abstract

Pseudoxanthoma elasticum (PXE) is a heritable multisystem ectopic calcification disorder. The gene responsible for PXE, *ABCC6*, encodes ABCC6, a hepatic efflux transporter regulating extracellular inorganic pyrophosphate (PPi), a potent endogenous calcification inhibitor. Recent studies demonstrated that in addition to the deficiency of plasma PPi, the activated DDR/PARP signaling in calcified tissues provides an additional possible mechanism of ectopic calcification in PXE. This study examined the effects of etidronate (ETD), a stable PPi analog, and its combination with minocycline (Mino), a potent inhibitor of DDR/PARP, on ectopic calcification in an *Abcc6^-/-^* mouse model of PXE. *Abcc6^-/-^* mice, at 4 weeks of age, before the development of ectopic calcification, were treated with ETD, Mino, or both for 18 weeks. Micro-computed tomography, histopathologic examination, and quantification of the calcium content in *Abcc6^-/-^* mice treated with both ETD and Mino revealed further reduced calcification than either treatment alone. The effects were associated with reduced serum alkaline phosphatase activity without changes in plasma PPi concentrations. These results suggest that ETD and Mino combination therapy might provide an effective therapeutic approach for PXE, a currently intractable disease.

## 1. Introduction

Pseudoxanthoma elasticum (PXE; OMIM no. 264800) is a heritable ectopic calcification disorder affecting the skin, eyes, and cardiovascular system [1]. While PXE is a late-onset, slowly progressive disease, it carries a high risk of morbidity due to the involvement of the retina, leading to loss of visual acuity and blindness. PXE can occasionally lead to early demise from vascular complications, including gastrointestinal bleeding, early myocardial infarction, and stroke. Cutaneous lesions consist of small, asymptomatic, yellowish papules or larger coalescent plaques, typically on the neck and the flexural areas. While the skin changes of PXE are mostly cosmetic, they are prevalent and signify the later developments of ocular and vascular involvements in the affected individuals, which result in substantial morbidity and mortality [2]. There currently is no effective treatment for PXE, and the disease slowly progresses after diagnosis.

Biallelic mutations in the gene encoding the hepatic efflux transporter ATP-binding cassette subfamily C member 6 (ABCC6) underlie PXE [3,4,5]. *ABCC6* mutations can also cause generalized arterial calcification of infancy type 2 (GACI2; OMIM no. 614473), a severe autosomal recessive disorder characterized by congenital calcification of arterial blood vessels [6,7]. The ABCC6 transporter mediates the release of ATP from hepatocytes into the blood circulation. Extracellularly, yet within the liver niche, ectonucleotide pyrophosphatase/phosphodiesterase 1 (ENPP1) converts released ATP into AMP and the mineralization inhibitor inorganic pyrophosphate (PPi) [8,9]. ANKH was also shown to mediate cellular efflux of ATP and contribute to PPi in plasma [10,11]. The ectopic calcification seen in PXE patients was initially thought to be a result of insufficient PPi generation in the circulation due to loss of ABCC6 in the liver [8]. As plasma PPi deficiency is considered a major determinant of ectopic calcification [12,13], therapies targeting the increase of PPi plasma concentrations have been shown to prevent the formation of these lesions in *Abcc6* knockout murine models of PXE [14,15]. Etidronate (ETD), a stable PPi analog with anti-calcification properties, partially prevented ectopic calcification in *Abcc6* knockout mice [16,17]. In a double-blinded clinical trial of adult PXE patients, ETD reduced subretinal neovascularization events compared to placebo [18] and significantly halted progression of vascular calcification except for the coronary arteries [19].

Recently, activation of oxidative stress and/or DNA damage response (DDR) pathways, in particular poly(ADP-ribose) polymerase 1 (PARP1) signaling at sites of ectopic calcification, was found to play an important role in vascular calcification [20]. The DDR/PARP pathway was also found to contribute to the pathogenesis of PXE [21,22]. Minocycline (Mino) recently emerged as a potent inhibitor of PARP, significantly reducing ectopic calcification in *Abcc6* knockout mice and *abcc6a* knockout zebrafish [21,22,23]. Mino also inhibited calcification of dermal fibroblasts cultured from PXE patients [21,22]. In these studies, inhibiting the local DDR/PARP signaling partially prevented ectopic calcification in PXE.

Identifying the possible mechanisms of PXE—both systemic and local factors contributing to ectopic calcification in PXE [21]—prompted us to develop novel therapies by targeting these pathways to arrest ectopic calcification completely. The metabolic nature of PXE is supported by the reduced circulating concentrations of PPi. On the other hand, local activation of DDR and PAR deposition triggers calcium hydroxyapatite deposition in peripheral connective tissues prone to ectopic calcification. Inhibition of the DDR/PARP pathway, either via genetic or pharmacological means, did not alter plasma PPi concentrations, suggesting that the systemic and local pathways do not intersect [21]. Despite significant progress in understanding the pathomechanisms from ABCC6 deficiency to ectopic calcification, PXE remains an intractable disease. In this study, we determined the effect of combined therapy of ETD and Mino by targeting the systemic and local pathways of ectopic calcification in PXE on reducing spontaneous ectopic calcification in *Abcc6^-/-^* mice.

## 2. Results

### 2.1. Combined ETD and Mino Treatment Reduced Ectopic Calcification in Abcc6^-/-^ Mice More Than Either Treatment Alone

The effects of combined ETD and Mino treatment on ectopic calcification in the *Abcc6^-/-^* mice were examined in an 18-week calcification prevention study (Table 1). The spontaneous ectopic calcification in *Abcc6^-/-^* mice is slowly progressive, with ectopic calcification occurring as early as 5–6 weeks postnatally. At 22 weeks of age, the untreated *Abcc6^-/-^* control mice developed robust and quantifiable calcification in the connective tissue dermal sheath of vibrissae in muzzle skin, a phenotypic hallmark in this mouse model of PXE [24]. We therefore initiated treatments in *Abcc6^-/-^* mice at 4 weeks of age, when ectopic calcification has not developed, and continued the treatments for 18 weeks. The untreated WT and *Abcc6^-/-^* mice served as negative and positive controls of ectopic connective tissue calcification.

**Figure 1:**
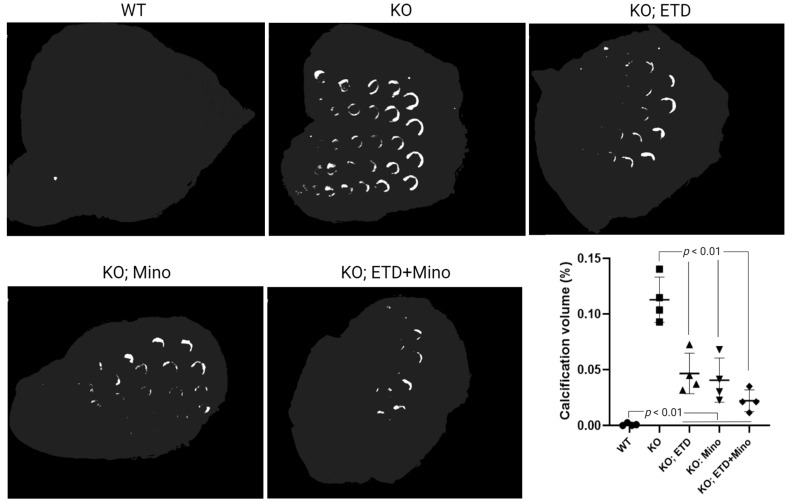
Ectopic calcification in the muzzle skin biopsies of *Abcc6^-/-^* mice analyzed by µCT scanning. The *Abcc6^-/-^* control mice developed extensive ectopic calcification of vibrissae in the muzzle skin. The WT mice were entirely negative for calcification in the muzzle skin. The *Abcc6^-/-^* mice treated with ETD or Mino demonstrated significantly decreased calcification compared to untreated *Abcc6^-/-^* control mice. The combined ETD and Mino treatment further reduced ectopic muzzle skin calcification. Quantitative analysis showed a significant reduction in calcification volume following treatments (bottom right panel). Values were expressed as mean ± SD; *n* = 4 mice per group.

**Figure 2:**
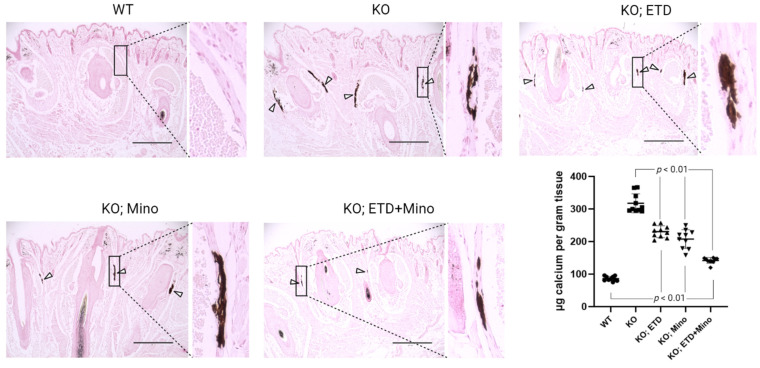
Ectopic calcification in the muzzle skin biopsies of *Abcc6^-/-^* mice analyzed by histology and calcium assay. Muzzle skin biopsies were collected and analyzed by von Kossa stains. The *Abcc6^-/-^* control mice developed robust ectopic calcification of the connective tissue dermal sheath of vibrissae in the muzzle skin. The WT mice were negative for calcification in the muzzle skin. The *Abcc6^-/-^* mice treated with ETD or Mino demonstrated significantly less calcification compared to *Abcc6^-/-^* control mice. Arrowheads indicate ectopic calcification. Enlarged boxed areas in each panel are shown on the right. Scale bar, 0.4 mm. The chemical quantification of calcium in the muzzle skin biopsies containing the dermal sheath of vibrissae is shown in the bottom right panel. Values were expressed as mean ± SD; *n* = 10 mice per group.

**Figure 3:**
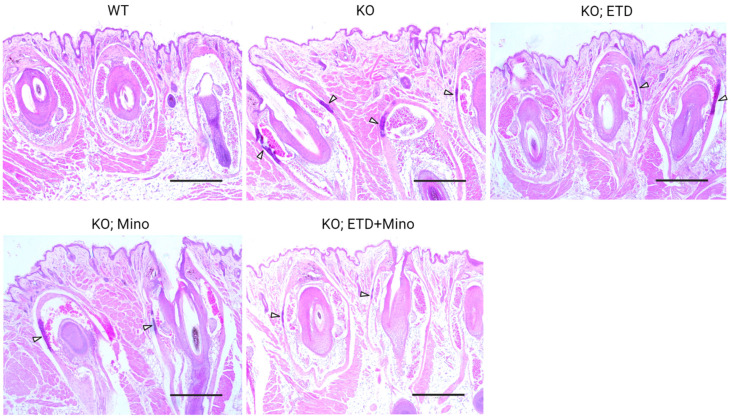
Ectopic calcification in the muzzle skin biopsies of *Abcc6^-/-^* mice analyzed by Hematoxylin and Eosin staining. The WT mice did not develop calcification in the muzzle skin. Extensive ectopic calcification was observed in the connective tissue dermal sheath of vibrissae in the muzzle skin of *Abcc6^-/-^* control mice. The *Abcc6^-/-^* mice treated with ETD or Mino demonstrated significantly less calcification compared to *Abcc6^-/-^* control mice. Arrowheads indicate ectopic calcification. Scale bar, 0.4 mm.

At 22 weeks of age, all mice were euthanized and analyzed. We measured calcification in the dermal sheath of vibrissae in the muzzle skin of *Abcc6^-/-^* mice using three independent assays. First, the calcification in one piece of muzzle skin was determined by µCT scanning (Figure 1). The results demonstrated extensive calcification in the muzzle skin biopsies of untreated *Abcc6^-/-^* control mice. In contrast, the wild-type (WT) mice did not have any calcification. Either ETD or Mino treatment alone significantly reduced ectopic calcification in the muzzle biopsies of *Abcc6^-/-^* mice. The combined ETD and Mino treatment further reduced ectopic calcification of muzzle skin, albeit residual ectopic calcification was still present. Volume measurement of calcified areas revealed that the calcification volume percentage in the ETD- and Mino-treated *Abcc6^-/-^* mice were reduced by 58.7% and 64.0%, respectively. The combined treatment reduced calcification volume by 80.3% compared to age-matched *Abcc6^-/-^* control mice (Figure 1). The severity of ectopic calcification was subsequently assessed by semi-quantitative histological evaluation (Figure 2). A calcium-specific von Kossa staining detected robust calcification in the dermal sheath of vibrissae in the untreated *Abcc6^-/-^* control mice. In contrast, the WT mice had no evidence of calcification. The *Abcc6^-/-^* mice receiving either ETD or Mino showed significantly reduced calcification. The combined ETD and Mino treatment reduced ectopic calcification more than either treatment alone. The Hematoxylin and Eosin staining showed similar results (Figure 3). Lastly, the efficacy of treatments was substantiated by measuring the calcium content in the other muzzle skin biopsy (Figure 2). The quantitative chemical assay of calcium showed that the calcium content in the ETD- and Mino-treated *Abcc6^-/-^* mice was significantly reduced by 37.3% and 47.4%, respectively. The combined ETD and Mino treatment reduced ectopic calcification by 76.0% compared to age-matched *Abcc6^-/-^* control mice. No significant differences were observed between male and female mice in any group.

### 2.2. Combined ETD and Mino Treatment Reduced Serum Alkaline Phosphatase Activity but Did Not Affect Plasma PPi Concentration in Abcc6^-/-^ Mice

**Figure 4:**
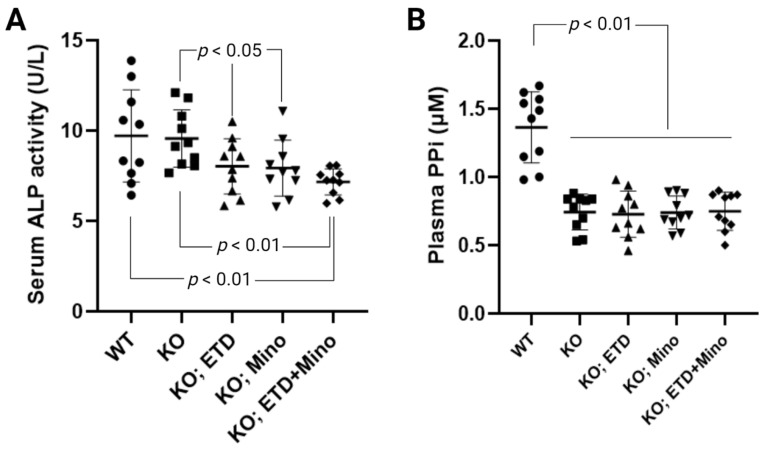
Biomarker response in *Abcc6^-/-^* mice treated with ETD, Mino, or both. (**A**) Serum alkaline phosphatase activity in *Abcc6^-/-^* mice. ETD, Mino, and combined treatment significantly reduced serum alkaline phosphatase activity. (**B**) Plasma PPi concentrations. The *Abcc6^-/-^* control mice had lower plasma PPi concentrations than WT mice. ETD, Mino, and combined treatment did not alter plasma PPi concentrations. Values were expressed as mean ± SD; *n* = 10 mice per group.

ETD reduces serum alkaline phosphatase activity in patients with Paget’s disease [25]. The anti-calcification effects of Mino were accompanied by reduced serum alkaline phosphatase activity; increased activities of this enzyme are known to promote ectopic calcification. To examine the mechanisms of ETD, Mino, or both on calcification prevention, we measured serum alkaline phosphatase activity in *Abcc6^-/-^* mice (Figure 4A). Serum alkaline phosphatase activity was similar in untreated WT and *Abcc6^-/-^* control mice. Compared to age-matched *Abcc6^-/-^* control mice, ETD and Mino treatment showed significantly reduced serum alkaline phosphatase activity, approximately 16.1% and 17.1%, respectively. The combined ETD and Mino treatment reduced serum alkaline phosphatase activity by 25.2%. To examine whether PPi homeostasis was affected, plasma concentrations of PPi were determined (Figure 4B). Consistent with previous findings [21,26], plasma PPi levels in the *Abcc6^-/-^* control mice were approximately 45% of that in the WT control mice. While ETD is a PPi analog, the PPi assay does not detect ETD, and thus, the *Abcc6^-/-^* mice treated with ETD had plasma PPi concentrations indistinguishable from the *Abcc6^-/-^* control mice. Mino or combined ETD and Mino did not change plasma PPi concentrations.

## 3. Discussion

To our knowledge, this is the first study that evaluated the effects of combined therapy with ETD and Mino on ectopic connective tissue calcification in *Abcc6^-/-^* mice, the gold standard mammalian PXE model. The effects were analyzed by three independent assays for ectopic calcification of the muzzle skin—µCT imaging, histopathology, and a quantitative chemical assay for calcium. ETD and Mino prevented ectopic muzzle skin calcification in *Abcc6^-/-^* mice by acting as a PPi analog and inhibiting local DDR/PARP signaling, respectively. Ectopic muzzle skin calcification in *Abcc6^-/-^* mice was further reduced by a combined ETD and Mino treatment. Although we do not exclude the possibility that additional mechanisms may explain the ectopic calcification in PXE, a treatment approach targeting plasma PPi deficiency and DDR/PARP activation in calcification-prone tissues is effective to reduce ectopic calcification in *Abcc6^-/-^* mice.

The anti-calcification effects of ETD and Mino in *Abcc6^-/-^* mice were accompanied by reduced serum alkaline phosphatase (ALP) activity; increased activities of this enzyme are known to promote ectopic calcification. We also found that the serum ALP activities were similar between the untreated WT and *Abcc6^-/-^* mice. The serum ALP activities in PXE patients were reported with conflicting results. One study reported elevated serum ALP levels in PXE patients (*n* = 18) compared to controls (*n* = 16) [27]. Another study reported similar serum ALP activities between PXE patients (*n* = 107) and healthy controls (*n* = 26) [28]. The reason for this discrepancy is unclear.

The results suggest that a combined therapy of ETD and Mino is superior to each treatment alone, with the potential to achieve a more complete arrest of ectopic calcification in patients with PXE and GACI2 carrying loss-of-function mutations in *ABCC6*. Clinical experience with ETD and Mino extends over more than 50 years, and both drugs demonstrate good long-term safety profiles with well-characterized pharmacokinetics. With specific relevance to PXE, ETD was recently shown to inhibit arterial calcification and subretinal neovascularization events in a double-blinded, single-center, one-year study consisting of 74 adult PXE patients [18,19]. Further studies are necessary to confirm the clinical benefits of a combination therapy.

## 4. Materials and Methods

### 4.1. Animal Studies

The *Abcc6^tm1JfK^* knock-out mouse was generated and described previously (this mouse is referred to as *Abcc6^-/-^*) [24]. The *Abcc6^-/-^* mice were bred congenic by at least ten backcrosses with wild-type (WT) C57BL/6J mice (The Jackson Laboratory, Bar Harbor, ME, USA). The study consisted of five groups of mice characterized by genotype and treatment, 10 mice per group (Table 1). The untreated WT and *Abcc6^-/-^* mice served as controls. These mice were fed a standard rodent diet (Lab Diet 5010; PMI Nutrition, Brentwood, MO, USA) and had free access to drinking water throughout the experiments. Some *Abcc6^-/-^* mice were fed a standard rodent diet supplemented with 0.984 mg/g etidronate (ETD), a dose corresponding to 240 mg/kg body weight/day that was previously shown to prevent ectopic muzzle skin calcification in *Abcc6^-/-^* mice [16]. Some *Abcc6^-/-^* mice were treated with 0.2 mg/mL minocycline (Mino) administered in the drinking water. This dose corresponds to 40 mg/kg body weight/day, assuming that a 20-g mouse consumes 4 mL of water per day. Some *Abcc6^-/-^* mice were treated with both ETD and Mino. The *Abcc6^-/-^* mice began treatment at 4 weeks of age, before ectopic calcification develops in these mice [24]. All mice were euthanized at 22 weeks of age, 18 weeks after treatment. All protocols were approved by the Institutional Animal Care and Use Committee of Thomas Jefferson University (Approval number 00669).

### 4.2. Serum Alkaline Phosphatase and Plasma PPi Measurements

At the end of the studies, whole blood was collected via cardiac puncture in all mice. To quantify PPi in plasma, whole blood was collected in heparin blood collection tubes (#365985; BD Diagnostics, Franklin Lakes, NJ, USA). Plasma was diluted with an equal volume of 50 mM tris acetate, pH 8.0, and depleted platelets using 30-kDa mass cutoff filters (#OD030C34; Pall Corporation, Exton, PA, USA). The PPi concentrations were measured in platelet-free plasma by an enzymatic reaction described previously [21,26]. The alkaline phosphatase activity was determined in serum using a colorimetric kit (#ab83369; Abcam, Waltham, MA, USA).

### 4.3. Analysis of Ectopic Calcification of Muzzle Skin

The left side muzzle skin biopsies were fixed in 10% phosphate-buffered formalin. Muzzle biopsies from four mice per group were subjected to micro-computed tomography (µCT) using a Skyscan 1275 µCT scanner with Skyscan software version 1.0.16 (Bruker Corporation, Billerica, MA, USA). Scans were acquired at source voltage 55 kV, source current 181 µA, and image pixel size 15 µm and saved in Tag Image File Format. Images were reconstructed using NRecon Reconstruction software 1.7.1, using an output range of (log) 0–0.055, and exported as a bitmap stack of virtual slices. CTAN and CTvox were used to isolate regions of interest and create 3D images. Ratios of calcified tissue volume/total tissue volume for each sample were calculated using CTAN’s 3D Analysis function and returned as default “Percent bone volume” values, which were computed as pixel^3^ of calcified tissue/pixel^3^ of total tissue; “bone” volume histogram range was set as 42–255 on grayscale index. All left side muzzle biopsies, ten mice per group, were then processed and embedded in paraffin, sectioned, and stained with Hematoxylin and Eosin and von Kossa. The von Kossa staining was done using a commercially available staining kit (#KTVKO; Mastertech Scientific KTVKO, Lodi, CA, USA). The kit contains 5% Silver Nitrate, 5% Sodium Thiosulfate, and Nuclear Fast Red Stain. Silver Nitrate serves as the primary stain, reacting with the anions and ultraviolet light to produce brown to black calcium precipitates against pink to red cytoplasm and nuclei, counterstained by Nuclear Fast Red. Calcium deposits appeared in approximately 30 min.

To quantify the amount of calcium, the right side muzzle skin biopsies, ten mice per group, were decalcified with 200 µL 1.0 mol/L HCl at room temperature for two days. The content of solubilized calcium was measured by a colorimetric assay kit (#0150-250; Stanbio Laboratory, Boerne, TX, USA). The calcium content in the muzzle biopsies was normalized to tissue weight.

### 4.4. Statistical Analysis

The results in different groups of mice were analyzed using ordinary one-way ANOVA. A *p* value < 0.05 is considered statistically significant. All statistical analyses were completed using Prism 9 software (GraphPad, San Diego, CA, USA).

## Figures and Tables

**Table 1:** Experimental groups of mice by genotype and treatment regimen.

Mice	Treatment ^†^	Delivery Route	Dose	No. of Mice (F + M) ^‡^	Treatment Starts > Treatment Ends
*Abcc6* ^+/+^	–	–	–	5 + 5	4 wk > 22 wk
*Abcc6^-/-^*	–	–	–	5 + 5	4 wk > 22 wk
*Abcc6^-/-^*	ETD	Oral (food mixture)	0.984 mg/g	5 + 5	4 wk > 22 wk
*Abcc6^-/-^*	Mino	Oral (drinking water)	0.2 mg/mL	6 + 4	4 wk > 22 wk
*Abcc6^-/-^*	ETD+Mino	Oral	ETD: 0.984 mg/g Mino: 0.2 mg/mL	5 + 5	4 wk > 22 wk

^†^ ETD, etidronate; Mino, minocycline. ^‡^ F, female; M, male.

## Data Availability

All data generated or analyzed during this study are included in this article.

## Abbreviations

etidronate (ETD), generalized arterial calcification of infancy (GACI), inorganic pyrophosphate (PPi), knock-out (KO), minocycline (Mino), pseudoxanthoma elasticum (PXE), wild-type (WT)
